# Tissue remodelling and increased DNA damage in patients with incompetent valves in chronic venous insufficiency

**DOI:** 10.1111/jcmm.16711

**Published:** 2021-06-20

**Authors:** Miguel A. Ortega, Oscar Fraile‐Martínez, Cielo García‐Montero, Leonel Pekarek, Miguel A. Alvarez‐Mon, Luis G. Guijarro, Maria del Carmen Boyano, Felipe Sainz, Melchor Álvarez‐Mon, Julia Buján, Natalio García‐Honduvilla, Ángel Asúnsolo

**Affiliations:** ^1^ Department of Medicine and Medical Specialities Faculty of Medicine and Health Sciences University of Alcalá Alcalá de Henares Spain; ^2^ Ramón y Cajal Institute of Healthcare Research (IRYCIS) Madrid Spain; ^3^ Cancer Registry and Pathology Department Hospital Universitario Principe de Asturias Alcalá de Henares Spain; ^4^ Unit of Biochemistry and Molecular Biology Department of System Biology University of Alcala Alcalá de Henares Spain; ^5^ Centro de Investigación Biomédica en Red de Enfermedades Hepáticas y Digestivas (CIBEREHD) Barcelona Spain; ^6^ Department of Surgery, Medical and Social Sciences Faculty of Medicine and Health Sciences University of Alcalá Alcalá de Henares Spain; ^7^ Angiology and Vascular Surgery Service Central University Hospital of Defence‐UAH Madrid Alcalá de Henares Spain; ^8^ Immune System Diseases‐Rheumatology Oncology Service and Internal Medicine University Hospital Príncipe de Asturias Alcalá de Henares Spain; ^9^ Department of Epidemiology and Biostatistics Graduate School of Public Health and Health Policy The City University of New York New York NY USA

**Keywords:** cellular damage, matrix remodelling, PARP, valve incompetence

## Abstract

Chronic venous insufficiency (CVI), in which blood return to the heart is impaired, is a prevalent condition worldwide. Valve incompetence is a complication of CVI that results in blood reflux, thereby aggravating venous hypertension. While CVI has a complex course and is known to produce alterations in the vein wall, the underlying pathological mechanisms remain unclear. This study examined the presence of DNA damage, pro‐inflammatory cytokines and extracellular matrix remodelling in CVI‐related valve incompetence. One hundred and ten patients with CVI were reviewed and divided into four groups according to age (<50 and ≥50 years) and a clinical diagnosis of venous reflux indicating venous system valve incompetence (R) (n = 81) or no reflux (NR) (n = 29). In vein specimens (greater saphenous vein) from each group, PARP, IL‐17, COL‐I, COL‐III, MMP‐2 and TIMP‐2 expression levels were determined by RT‐qPCR and immunohistochemistry. The younger patients with valve incompetence showed significantly higher PARP, IL‐17, COL‐I, COL‐III, MMP‐2 and reduced TIMP‐2 expression levels and a higher COL‐I/III ratio. Young CVI patients with venous reflux suffer chronic DNA damage, with consequences at both the local tissue and systemic levels, possibly associated with ageing.

## 1 INTRODUCTION

Chronic venous insufficiency (CVI), in which blood return to the heart is impaired, has a high prevalence and incidence worldwide.^1^, ^2^ There is a higher incidence of CVI in women than men,^3^ and CVI has been related to factors such as pregnancy, hormones, lifestyle, family history, metabolic abnormalities and ageing.^4^, ^5^ In western Europe, medical care costs for CVI patients have been estimated at 600‐900 million Euros, accounting for 2% of total expenditures.^6^ To understand CVI, it must be viewed as a cause of venous hypertension whose most characteristic clinical manifestation is the appearance of varicose veins.^3^, ^7^ A common complication of CVI is the development of valve dysfunction or incompetence that produces blood reflux, which aggravates venous hypertension and increases hydrostatic pressure.^8^

CVI is a complex disease in which the vein wall undergoes changes, although the pathological mechanisms involved in the homeostatic imbalance of CVI remain unclear. Alterations in the extracellular matrix (ECM) have been shown to be essential in this venous disease.^9^, ^10^ Collagen fibres play an important role by participating in the homeostasis of vascular cells (endothelial cells, muscle cells and fibroblasts).^11^, ^12^ In the vascular system, collagens I (COL‐I) and III (COL‐III) are found in greater proportions and are modified depending on the pathology.^13^ The ECM undergoes permanent remodelling involving the degradation and synthesis of new proteins by proteases such as metalloproteinases (MMPs).^14^ The structural and functional diversity of MMPs rival those of the collagen superfamily, and MMP‐2 has a prominent role in vascular remodelling.^15^, ^16^, ^17^ The proteolytic activity of MMP‐2 is regulated at three levels: first—via expression and secretion; second—through pro‐enzyme activation and third—through inhibition by tissue inhibitors of MMPs (TIMPs). This regulation serves to maintain a delicate balance.^18^, ^19^ Changes in the vein wall lead to an inflammatory process in which pro‐inflammatory cytokines trigger cellular responses.^20^, ^21^, ^22^, ^23^, ^24^ Recent studies have revealed the presence of an oxidative stress environment that has a direct effect on signal transduction pathways in patients with CVI, with consequences at both the systemic and vein wall levels.^25^, ^26^, ^27^ Moreover, metabolic and chronic disorders can lead to DNA damage, inducing an increase in the activity of repair molecules such as poly(ADP‐ribose) polymerase (PARP).^28^, ^29^

The aims of this study were to examine the consequences of CVI on the venous wall for a better understanding of the pathophysiological consequences for analysing the (a) possible alterations in PARP expression, (b) the role of pro‐inflammatory cytokines such as IL‐17 and (c) ECM remodelling in CVI patients with valve incompetence (venous reflux). These factors were also examined in relation to patient age.

## 2 PATIENTS AND METHODS

### 2.1 Study population

One hundred and ten patients were divided by age (cut‐off, 50 years) and the presence (R) or absence (NR) of a clinical diagnosis of venous reflux (an indicator of venous system valve incompetence). The following study groups were established as follows: NR, n = 29, 50.0 [31.0‐79.0] years; NR<50, n = 13, 38.0 [31.0‐48.0] years; NR≥50, n = 16, 62.5 [50.0‐79.0] years; R, n = 81, 51.0 [22.0‐79.0] years; R < 50, n = 32, 35.0 [22.0‐48.0] years and R ≥ 50, n = 49, 62.0 [50.0‐79.0] years. The study cohort was selected according to the following criteria. The patients were divided according to age, fifty‐year cut‐off point based on the scientific and clinical‐medical evidence observed in the scientific literature.

#### 2.1.1 Inclusion criteria

women and men diagnosed with CVI with or without venous reflux in the great saphenous vein; BMI ≤25; signed informed consent; commitment to follow‐up during pre‐ and post‐operative periods; and permission for tissue collection for gene and pathological anatomy studies.

#### 2.1.2 Exclusion criteria

patients with venous malformations or arterial insufficiency; patients who did not provide their medical history; patients with a pathology affecting the cardiovascular system (eg infectious diseases, diabetes, dyslipidaemia, hypertension); patients with toxic habits [tobacco (≥1 cigarette a day), alcohol (≥1 unit a day) or drugs (cannabis, heroin, cocaine, amphetamins)] and patients reluctant to commit to follow‐up. This study was conducted in accordance with the basic ethical principles of autonomy, beneficence, non‐maleficence and distributive justice. The study protocol was in line with the standards of Good Clinical Practice and the principles set out in the most recent Declaration of Helsinki (2013) and Oviedo Convention (1997). The patients were duly informed, and each was asked to provide written informed consent. The study was approved by the Ethics Committee of Clinical Investigations of the Central University Hospital of Defense Gómez‐Ulla‐UAH (PI18/00912‐03‐37/17).

### 2.2 Diagnosis by imaging

The patients were performed using a 7.5‐Mz M Turbo Transducer Echo‐Doppler (SonoSite). The lower limb examination was performed in the standing position, with one leg in external rotation and the contralateral leg supported. The exam included the great saphenous axis from the inguinal region to the femoral ankle and vein. The saphenous vein and popliteal vein were also evaluated in the standing position, with the patient facing away from the examiner and with the leg unloaded, as described by Ortega et al^27^ Venous reflux was defined as a reflux time longer than 0.5 seconds. Saphenectomy was indicated in all patients. In the patients with NR, vascular compressive syndrome was the surgical indication. Classification of Chronic Venous Insufficiency was performed according to the Classification System for Chronic Venous Disorders (CEAP) includes the full spectrum of morphologic and functional abnormalities of the venous system, from telangiectasies to venous ulcers. Patients have been a CEAP classification C1 or higher (Table S1).

### 2.3 Collection of vein tissue samples

At the end of the saphenectomy operation, the total saphenous arch was removed for analysis and placed into two sterile tubes, one with Minimum Essential Medium (MEM) with 1% antibiotic/antimycotic (both from Thermo Fisher Scientific) and another containing RNAlater^®^ Solution (Ambion). In all cases, the samples were transported under refrigeration within four hours of collection. In the laboratory, the samples were processed under sterile conditions under a Telstar AV 30/70 Müller 220 Hz class II laminar flow hood (Group Telstar SA). The samples preserved in MEM were destined for histological studies; they were washed/hydrated several times with MEM without antibiotics to remove blood cells and cut into fragments that were placed in F13 fixative (60% ethanol, 20% methanol, 7% polyethylene glycol and 13% distilled H_2_O) for future studies. The samples placed in RNAlater^®^ were stored in 1 mL of this solution at −80℃ until further processing for gene expression analysis.

### 2.4 Immunohistological studies

After the necessary time in the fixation solution (10 days), the samples were dehydrated and processed according to standardized protocols.^25^ Then, paraffin blocks were made using moulds. Once the paraffin solidified, a rotation microtome HM 350 S (Thermo Fisher Scientific) was used to cut 5‐μm‐thick sections, which were stretched in a hot water bath and collected on glass slides coated with 10% poly‐L‐lysine to facilitate tissue bonding. Antigen‐antibody reactions were detected using the avidin‐biotin complex (ABC) method with specific primary (Table 1A) and secondary antibodies (Table 1B) and chromogen peroxidase or alkaline phosphatase according to the protocol published by Ortega et al^25^ The slides were incubated with the avidin‐peroxidase conjugate ExtrAvidin^®^‐Peroxidase (1/200; Sigma‐Aldrich) for 1 hour at room temperature. For avidin‐alkaline phosphatase conjugates, ExtrAvidin^®^‐Alkaline Phosphatase (1/200; Sigma‐Aldrich) was used for 1 hour at room temperature. Avidin‐peroxidase conjugate signals were developed by incubation with the chromogenic substrate diaminobenzidine (DAB Kit, SK‐4100; Vector) for 15 minutes (the signal was monitored under the microscope). The chromogenic substrate was prepared immediately before development: 5 mL of distilled water, two drops of buffer, four drops of DAB and two drops of hydrogen peroxide, according to DAB Kit. This technique allows for brown staining. In the case of the avidin‐alkaline phosphatase conjugate, the signal was developed with the alkaline chromogenic substrate for 15 minutes (monitoring the signal under the microscope). The chromogenic substrate was prepared immediately before development by adding 10 mL of PBS to 10 mg of α‐naphthol AS‐BI phosphate, 10 mg of Fast red, and 100 μL of 0.1 mol/L levamisole. For all immunohistochemical studies, sections of the same tissue incubated in blocking solution without the primary antibody were used as negative controls.

**TABLE 1:** Primary (A) and secondary (B) antibodies with dilution factors and protocol specifications in the immunohistological studies

Antigen	Species	Dilution	Provider	Protocol specifications
Primary (A)				
PARP	Mouse	1:1000	Abcam (ab110915)	10 mmol/L Sodium citrate Ph = 6 before incubation with blocking solution
IL‐17	Rabbit	1:250	Abcam (ab79056)	Triton 0.1% in PBS, 10 min, before incubation with blocking solution
COL‐I	Mouse	1:400	Sigma‐Aldrich (C 2456)	‐
COL‐III	Mouse	1:500	Abcam	‐
MMP‐2	Mouse	1:1000	NeoMarkers (CA‐4001)	‐
TIMP‐2	Mouse	1:50	Abcam (ab74216)	‐
secondary (B)				
IgG (Mouse)	Goat	1:300	Sigma (F2012/045K6072)	‐
IgG (Rabbit)	Mouse	1:1000	Sigma (RG‐96/ B5283)	‐

### 2.5 Sirius red staining

This technique distinguishes immature collagen (COL‐III), which appears yellowish‐green, from mature collagen (COL‐I), which appears reddish. Staining is observed under a dark‐field microscope. The following protocol was utilized as follows: (a) Sirius red staining for 30 minutes; (b) wash with running water; (c) dehydration in 100% alcohol for 5 minutes; (d) clearance of the sections in xylol for 10 minutes and (e) assembly with Cytoseal™.

### 2.6 Analysis of gene expression using qRT‐PCR

RNA was extracted from the samples collected in RNAlater^®^ using the guanidine‐phenol‐chloroform isothiocyanate method,^25^ and RNA samples were always maintained on ice. To synthesize complementary DNA (cDNA) via reverse transcription, RNA samples were diluted to 50 ng/μL; then, 4 μL of diluted RNA was mixed with 4 μL of oligo‐dT solution at 0.25 µg/µL (Thermo Fisher Scientific A.) and incubated at 65℃ for 10 minutes in a dry bath (AccuBlock™, Labnet International Inc.) to denature the RNA. Next, the samples were placed on ice, and 10 μL of a reverse transcription mixture was added to each sample following established protocols.^27^ Reverse transcription was performed to synthesize cDNA using a G‐Storm GS1 thermocycler (G‐Storm, Somerset, UK) at 37℃ for 1 hour and 15 minutes, 70℃ for 15 minutes (to denature the reverse transcriptase enzyme), and a gradual decrease to 4℃. To verify the absence of genomic DNA contamination in the total RNA samples, negative control samples were run in parallel; in these samples, M‐MLV RT was replaced with DNase‐ and RNase‐free water. The generated cDNA was diluted at 1:20 using DNase‐ and RNase‐free water and stored at −20℃ until use. The qPCR was performed to quantify the levels of cDNA corresponding to the genes of interest in each sample. Specific primers were designed de novo for the studied genes (Table 2) using Primer‐BLAST and AutoDimer.^27^ The constitutively expressed glyceraldehyde 3‐phosphate dehydrogenase (GAPDH) gene was used to normalize the results. qPCR was carried out in a StepOnePlus™ System (Thermo Fisher Scientific), and the relative standard curve method was used. For each sample, 5 μL of the sample diluted 1/20 was mixed with 10 μL of iQ™ SYBR^®^ Green Supermix (Bio‐Rad Laboratories), 1 μL of forwarding primer, 1 μL of reverse primer and 3 μL of DNase‐ and RNase‐free water, for a total reaction volume of 20 μL in a 96‐well MicroAmp^®^ plate (Thermo Fisher Scientific). Fluorescence was detected at the end of each amplification cycle and at each step of the dissociation curve. The data obtained for each gene were compared to a standard curve based on serial dilutions of a mixture of the study samples included in each plate according to the expression of GADPH. Gene expression units have been expressed as relative quantity mRNA (RQ). All the assays were performed in duplicate (Table S2).

**TABLE 2:** Sequences of the primers and their binding temperatures in the RT‐qPCR studies

Gene	Sequence Fwd (5′→3′)	Sequence Rev (5′→3′)	Temperature
GADPH	GGA AGG TGA AGG TCG GAG TCA	GTC ATT GAT GGC AAC AAT ATC CAC T	60^°^C
PARP	CCA GGA TGA AG AGG CAG TGA AG	TTC TGA AGG TCG ATC TCA TAC TCC	58^°^C
IL‐17	CAA CCG ATC CAC CTC ACC	AGC CCA CGG ACA CCA GTA	61^°^C
COL‐I	CCA TGT GAA ATT GTC TCC CA	GGG GCA AGA CAG TGA TTG AA	60^°^C
COL‐III	GAC TTC CAA GAC CTC TTT	CCA CAA GGA TTA CAA GGC TTG	62^°^C
MMP‐2	ATA ACC TGG ATG CCG TCG TG	CTT CAC GCT CTT CAG ACT TTG G	60^°^C
TIMP‐2	TCT GGA AAC GAC ATT TAT GG	GTT GGA GGC CTG CTT ATG GG	61^°^C

### 2.7 Statistical analysis and interpretation of the results

For statistical analyses, GraphPad Prism^®^ 5.1 software was used with Mann‐Whitney *U* test. The data are presented as the median with interquartile range (IQR). Significance was set at *P* < .05 (*) and *P* < .005 (**). For each patient in the established groups, five sections and 10 fields per section were randomly selected and examined. Patients were characterized as positive when the average marked area in the sample analysed was greater than or equal to 5% of the total area, following the anatomical‐pathological protocol of Cristóbal et al^30^ In each sample, immunohistochemical staining was scored using the following scale: 0‐1, minimum staining (0%‐25%); 2, moderate staining (25%‐65%); 3, strong staining (65%‐100%). This procedure is a minimal modification of the immunoreactive score (ISR score).^30^ The preparations were examined under a Zeiss Axiophot light microscope (Carl Zeiss) equipped with an AxioCam HRc digital camera (Carl Zeiss).

## 3 RESULTS

### 3.1 Patients with valve incompetence show elevated PPAR expression in the vein wall

**FIGURE 1:**
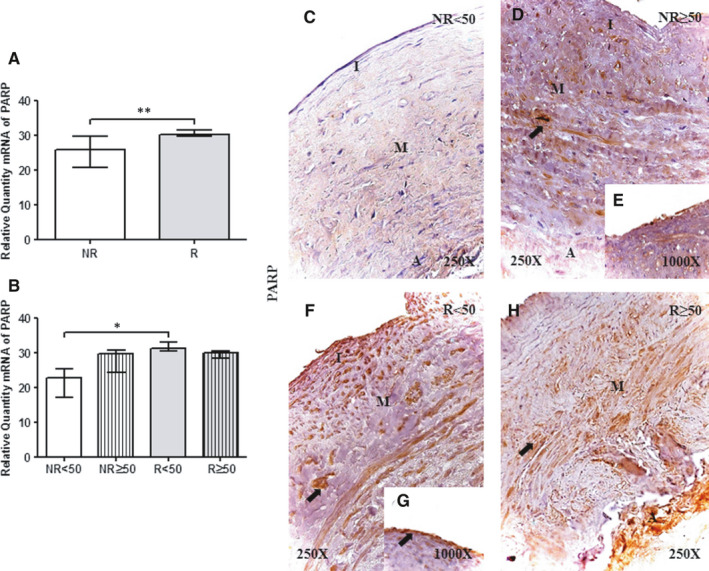
A and B, PARP mRNA expression levels in the vein wall in the different study groups as determined by RT‐qPCR. C‐H. Images of PARP protein in the vein wall of patients in the different study groups. NR<50, patients younger than 50 y without reflux; NR≥50, patients without reflux aged 50 y or older; R < 50, patients younger than 50 y with reflux; R ≥ 50, patients with reflux aged 50 y or older; I, tunica intima; M, tunica media; A, tunica adventitia. The arrow indicates PARP protein expression in the vein wall. *P* < .05 (*) and *P* < .005 (**).NR (n = 29) [NR<50(n = 13); NR≥50 (n = 16)]; R (n = 81) [R < 50 (n = 32); R ≥ 50 (n = 49)]. For statistical analyses, was used Mann‐Whitney *U* test

The qRT‐PCR showed a significant increase in PARP gene expression in samples with venous reflux (NR = 25.89 [15.39‐31.44], R = 30.33[20.77‐39.18] **P = .007; Figure 1A). When considering age, the R < 50 group had the highest PARP expression levels, which were significantly different from those in the NR < 50 group [*P = .01; NR < 50 = 22.84 [15.39‐25.88], NR ≥ 50 = 29.76 [19.48‐31.44], R < 50 = 31.25 [20.77‐38.74], R ≥ 50[29.92[26.13‐39.18]; Figure 1B and Table. S2A)).

The immunohistochemical analysis of PPAR protein expression showed that PPAR was present in 100% (n = 81) of R patients as opposed to 75% (n = 21) of NR patients. Considering patient age, all the patients showed PPAR expression except those in the NR<50 group, in which the percentage of positive expression was 38.46% (n = 5). Observations of the distribution of PPAR expression in the vein wall enabled characterization based on its presence in the three tunics in the different study groups (Figure 1). PPAR was strongly expressed in smooth muscle cells of the tunica media (Figure 1, arrow). Notably, R < 50 and NR≥50 patients showed intense expression in the tunica intima in the venous endothelium (Figure 1E,G). Immunoreactive score was higher in R < 50 patients according to ISR score (0.00[0.00‐1.50] NR < 50, 1.00[0.50‐2.50] NR ≥ 50, 3.00[1.00‐3.00] R < 50 and 2.00[0.50‐3.00] R ≥ 50).

### 3.2 Valve incompetence causes an increase in the expression of pro‐inflammatory cytokines (IL‐17)

**FIGURE 2:**
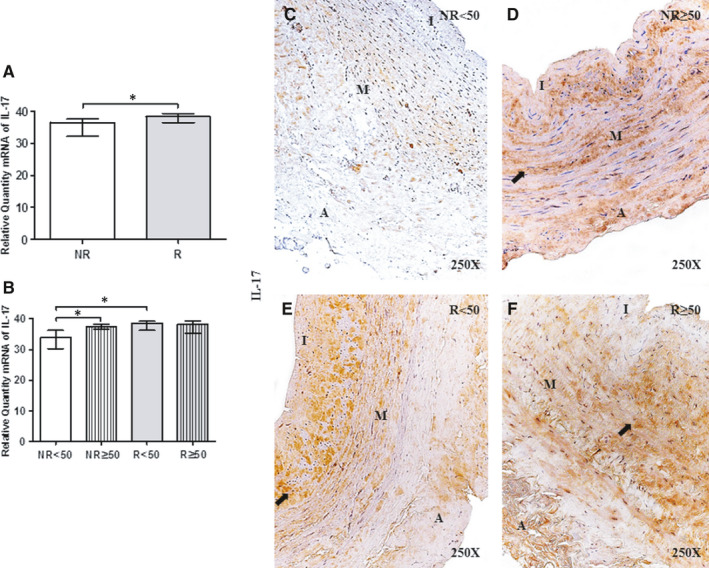
A and B, IL‐17 mRNA expression levels in the vein wall of patients in the different study groups as determined by RT‐qPCR. C‐F. Images of IL‐17 protein in the vein wall of patients in the different study groups. NR<50, patients younger than 50 y without reflux; NR≥50, patients without reflux aged 50 y or older; R < 50, patients younger than 50 y with reflux; R ≥ 50, patients with reflux aged 50 y or older; I, tunica intima; M, tunica media; A, tunica adventitia. The arrow indicates IL‐17 protein expression in the vein wall. *P* < .05 (*).NR (n = 29) [NR<50(n = 13); NR≥50 (n = 16)]; R (n = 81) [R < 50(n = 32); R ≥ 50 (n = 49)]. For statistical analyses, was used Mann‐Whitney *U* test

An analysis of pro‐inflammatory cytokines revealed a significant increase in IL‐17 gene expression in R patients compared with NR patients (NR = 36.52[27.45‐37.05], R = 38.36[29.45‐39.81]*P =.01; Figure 2A). Regarding patient age, R < 50 patients showed a significant increase in IL‐17 expression compared with NR<50 patients (**P = 0008) and in NR group (**P =.007); no significant differences were observed among the other study groups [(NR<50 = 33.84 [27.45‐37.05], NR≥50 = 37.42 [32.00‐38.80], R < 50=38.485 [29.45‐39.81], R ≥ 50[38.24] [30.25‐39.78]; Figure 2B and Table. S2)].

IL‐17 protein expression was positive in 100% of R patients (n = 81) but only 82.76% of NR patients (n = 24). In the NR<50 group, 38.46% (n = 5) of patients had positive IL‐17 expression. IL‐17 localization in the vein wall was uniform in all the study groups, and IL‐17 was detected in the three tunics. In addition, the presence of IL‐17 in the ECM and venous endothelium was noted (Figure 2C‐F). Interestingly, R patients showed particularly intense IL‐17 expression in smooth muscle cells (Figure 2E,F). Immunoreactive score was higher in R < 50 patients according to ISR score (0.00[0.00‐1.00] NR < 50, 1.00[0.50‐2.00] NR ≥ 50, 2.00[0.50‐3.00] R < 50 and 1.50[1.00‐3.00] R ≥ 50).

### 3.3 The vein wall of patients with valve incompetence undergoes collagen fibre remodelling

Extracellular matrix remodelling was investigated by analysing COL‐I and COL‐III expression levels and ratios using Sirius red staining.

**FIGURE 3:**
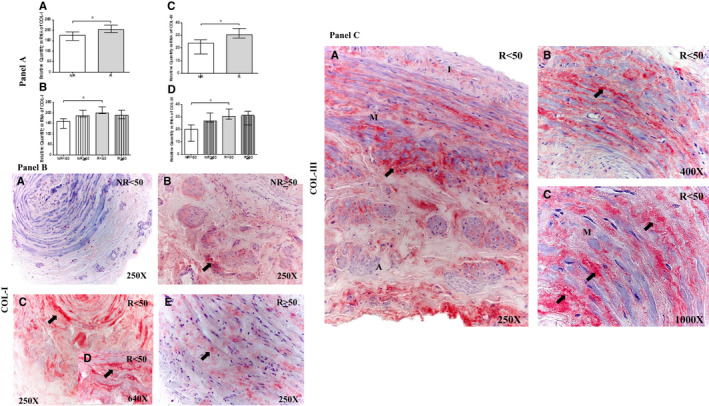
Panel A, Expression levels of Collagen Type I (COL‐I) mRNA (A and B) and Collagen Type III (COL‐III) mRNA (C‐D) in the different study groups. Panel B. Images of the protein expression (COL‐I) in the venous wall of the different study groups (A‐E). Panel C. Images of the protein expression (COL‐III) in the venous wall of the different study groups (A‐C). NR<50, patients younger than 50 y without reflux; NR≥50, patients without reflux aged 50 y or older; R < 50, patients younger than 50 y with reflux; R ≥ 50, patients with reflux aged 50 y or older; I, tunica intima; M, tunica media; A, tunica adventitia. The arrow indicates COL‐I and COL‐III protein expression in the vein wall. *P* < .05. asterisk = green colouration. NR (n = 29) [NR<50 (n = 13); NR≥50 (n = 16)]; R (n = 81) [R < 50(n = 32); R ≥ 50 (n = 49)]. For statistical analyses, was used Mann‐Whitney *U* test

First, COL‐I gene expression was significantly increased in R patients compared with NR patients (NR = 176.98[127.06‐214.19], R = 202.29[171.26‐240.78] *P = .03; Figure 3 Panel A. (A and B). In the groups categorized by age, COL‐I expression was significantly increased in R < 50 patients compared with NR patients (*P = .02); no significant differences were observed among the other study groups [(NR<50 = 158.29 [127.06‐172.81], NR≥50 = 184.81 [181.14‐214.19], R < 50 = 200.97 [188.89‐240.79], R ≥ 50 = 189.15[171.26‐114.55]; Figure 3 Panel A. (B) and Table. S2.C)].

Second, COL‐III gene expression was similarly significantly increased in R patients (NR = 23.76[10.40‐26.67], R = 30.59[23.37‐39.16], *P = .01; Figure 3 Panel A. (C)). Moreover, considering age, the R < 50 group showed a significant increase in COL‐III expression(*P = .02); no significant differences were observed among the other study groups [(NR < 50 = 19.94 [10.40‐23.76], NR ≥ 50 = 26.670 [25.87‐33.08], R < 50 = 30.20 [27.62‐39.16], R ≥ 50 = 31.12[23.37‐35.73]; Figure 3 Panel A. (D) and Table. S2.D)]. Moreover, the levels of COL‐I were much higher than those of COL‐III.

Among NR patients, 68.97% (n = 20) showed positive COL‐I protein expression, as opposed to 96.30% (n = 78) of R patients. When considering age, the NR<50 group had the lowest percentage of COL‐I expression (NR<50, 38.46%, n = 5; NR≥50, 93.75%, n = 15; R < 50, 96.78%, n = 31; R ≥ 50, 95.92%, n = 47). COL‐I expression was not uniform among the study groups; patients with R had greater COL‐I expression in the three tunics of the vein wall (Figure 3 Panel B. (C and D)). Notably, R < 50 patients appeared to exhibit a cross‐linked (collagen does not present in a linear way) COL‐I expression pattern (Figure 3 Panel B. (D), arrow). Immunoreactive score was higher in R < 50 patients (0.00[0.00‐1.00] NR < 50, 1.00[0.00‐2.00] NR ≥ 50, 2.50[0.00‐3.00] R < 50 and 1.50[0.00‐3.00] R ≥ 50).

For COL‐III protein, the expression percentage was 62.07% (n = 18) in NR patients and 82.72% (n = 67) in R patients. When considering age, the positive COL‐III expression percentage was 100% (n = 32) in R < 50 patients (NR < 50, 30.77%, n = 4; NR ≥ 50, 87.50%, n = 14; R ≥ 50, 71.43%, n = 35). Among patients with CVI, those in the R < 50 group showed COL‐III protein expression with a cross‐linking pattern in the three tunics of the vein wall (Figure 3 Panel C). (A, B and C). Immunoreactive score was higher in R < 50 patients according to ISR score (0.00[0.00‐0.50] NR<50, 1.00[0.00‐1.50] NR≥50, 2.00[0.50‐3.00] R < 50 and 1.25[0.00‐2.50] R ≥ 50).

### 3.4 Young patients with valve incompetence have an altered COL‐I/COL‐III ratio

**FIGURE 4:**
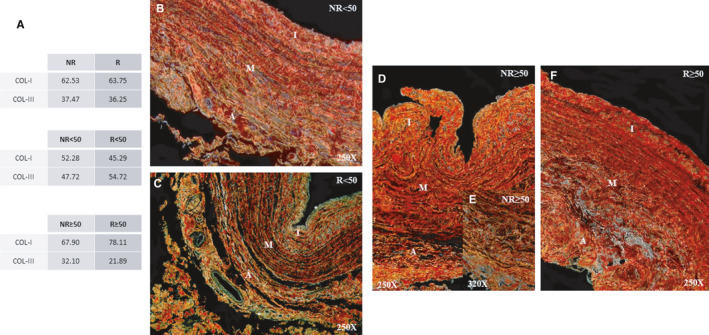
A, Percentage of patients with expression of Collagen Type I (COL‐I) and Collagen Type III (COL‐III) with Sirius Red technique. (orange‐red = COL‐I, yellow‐green = COL‐III). B‐E, Images of histological staining of collagen fibres with Sirius red in the venous wall of the different study groups. NR<50, patients younger than 50 y without reflux; NR≥50, patients without reflux aged 50 y or older; R < 50, patients younger than 50 y with reflux; R ≥ 50, patients with reflux aged 50 y or older; I, tunica intima; M, tunica media; A, tunica adventitia. NR (n = 29) [NR<50 (n = 13); NR≥50 (n = 16)]; R (n = 81) [R < 50 (n = 32); R ≥ 50 (n = 49)]. For statistical analyses, was used Mann‐Whitney *U* test

Through histological Sirius red staining and subsequent observation under polarized light, COL‐I/III coexpression in the vein wall was revealed. In general, the COL‐I/III ratio did not vary between NR and R patients, with both groups expressing high levels of COL‐I (Figure 4A). Considering age, there is a rational explanation for why R < 50 patients have a higher proportion of COL‐III; in histological sections of tissues from these patients, a clear predominance of the characteristic green colouration of this type of collagen can be observed with high intensity (Figure 4C. asterisk). In contrast, samples from the other study groups exhibited a yellow‐red wall (Figure 4B‐F).

### 3.5 Young patients with valve incompetence show elevated MMP‐2 expression and reduced TIMP‐2 expression

**FIGURE 5:**
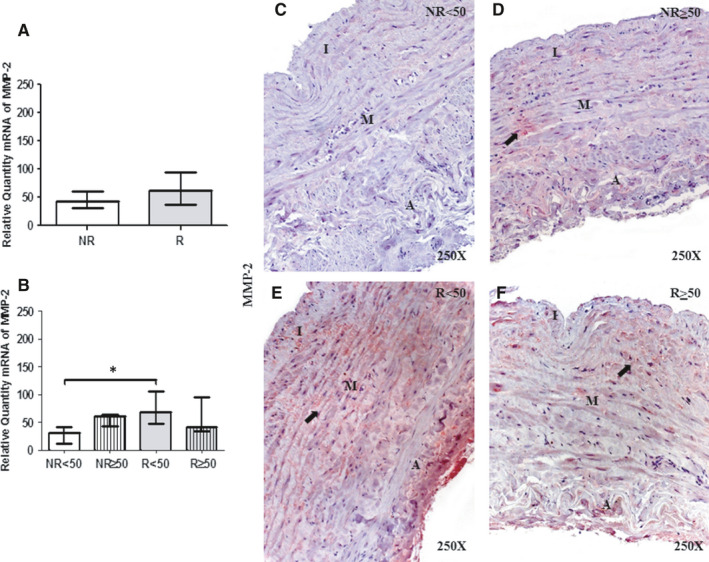
A and B, Levels of expression of MMP‐2 mRNA in the venous wall in the different study groups using the RT‐qPCR technique. C‐F. Images of the protein expression in the venous wall of the different study groups. NR<50, patients younger than 50 y without reflux; NR≥50, patients without reflux aged 50 y or older; R < 50, patients younger than 50 y with reflux; R ≥ 50, patients with reflux aged 50 y or older; I, tunica intima; M, tunica media; A, tunica adventitia. The arrow indicates MMP‐2 protein expression in the vein wall. *P* < .05 (*). Arrow = protein expression of MMP‐2 in the venous wall. *P* < .05 (*).NR (n = 29) [NR<50 (n = 13); NR≥50 (n = 16)]; R (n = 81) [R < 50 (n = 32); R ≥ 50 (n = 49)]. For statistical analyses, was used Mann‐Whitney *U* test

MMP‐2 gene expression was not significantly different between R and NR patients (NR = 42.68[5.41‐64.52], R = 61.53[32.41‐140.37], *P* = .10). When considering age, a significant increase in MMP‐2 expression was observed in R < 50 patients compared with NR < 50 patients (*P = .01); no significant differences were observed among the other study groups [(NR < 50 = 30.27 [5.41‐45.27], NR ≥ 50 = 60.00 [42.68‐64.52], R < 50=68.51 [37.04‐140.37], R ≥ 50 = 41.30[32.41‐129.79]; Figure 5A and Table. S2.E)].

MMP‐2 protein expression analysis showed a percentage of positive expression of 51.72% (n = 15) in NR patients and 62.96% in R patients. When considering age, the highest percentage of positive expression was observed in R < 50 patients (NR < 50, 38.46%, n = 5; NR≥50, 65.50%, n = 10; R < 50, 78.13%, n = 25; R ≥ 50, 53.06%, n = 26). Histological sections showed that MMP‐2 was distributed in the ECM of the three tunica, as well as in the intensity cytoplasm of smooth muscle cells (Figure 5C‐F). Notably, the expression was higher in the R < 50 group than in the other study groups (Figure 5E). Immunoreactive score was higher in R < 50 patients (0.00[0.00‐0.50] NR < 50, 0.50[0.00‐1.50] NR ≥ 50, 1.50[0.00‐2.00] R < 50 and 1.00[0.00‐1.50] R ≥ 50).

**FIGURE 6:**
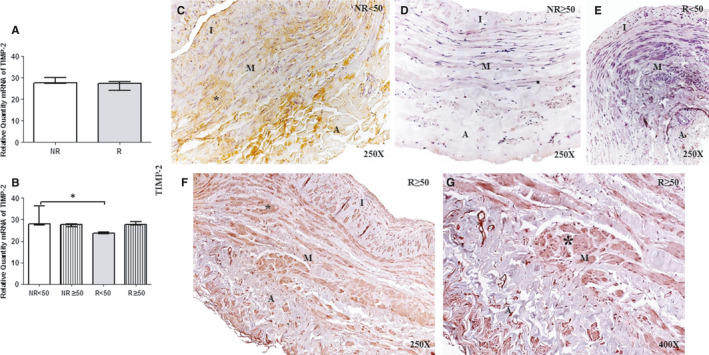
A and B, Levels of expression of TIMP‐2 mRNA in the venous wall in the different study groups using the RT‐qPCR technique. C‐G. Images of the protein expression in the venous wall of the different study groups. NR<50, patients younger than 50 y without reflux; NR≥50, patients without reflux aged 50 y or older; R < 50, patients younger than 50 y with reflux; R ≥ 50, patients with reflux aged 50 y or older; I, tunica intima; M, tunica media; A, tunica adventitia. The arrow indicates TIMP‐2 protein expression in the vein wall. *P* < .05 (*).NR (n = 29) [NR<50 (n = 13); NR≥50 (n = 16)]; R (n = 81) [R < 50 (n = 32); R ≥ 50 (n = 49)]. For statistical analyses, was used Mann‐Whitney *U* test

Conversely, TIMP‐2 gene expression was significantly decreased in R < 50 patients compared with NR < 50 patients (*P = .01); no significant differences were observed among the other study groups [(NR < 50 = 28.09 [27.54‐36.33], NR ≥ 50 = 27.63 [26.58‐27.93], R < 50 = 23.72[23.45‐24.27], R ≥ 50 = 27.73[26.52‐31.45]; Figure 6B and Table. S2.F)]. No significant differences were observed between NR and R patients (NR = 27.78[26.58‐36.33], R = 27.41[23.45‐31.45, *P* = .291]). The percentage of TIMP‐2 protein expression was 51.72% (n = 15) in NR patients and 48.15% (n = 39) in R patients, with the lowest percentage in R < 50 patients (NR < 50, 76.92%, n = 10; NR ≥ 50, 31.25%, n = 5; R < 50, 28.12%, n = 9; R ≥ 50, 61.22%, n = 30). Histological analyses showed that TIMP‐2 extended through the three tunica of the vein wall in the patients with positive expression. Remarkably, in NR < 50 patients, TIMP‐2 expression appeared as accumulations (Figure 6C, asterisk). In R ≥ 50 patients, high‐intensity TIMP‐2 expression was present in the three tunics of the vein, characteristic of findings in smooth muscle cells (Figure 6F,G). Patients in the other groups had mild‐intensity TIMP‐2 expression, and the majority of the samples were negative (Figure 6). Immunoreactive score was lower in R < 50 patients (2.00[0.00‐3.00] NR < 50, 0.00[0.00‐2.00] NR ≥ 50, 0.00[0.00‐1.00] R < 50 and 0.00[0.00‐2.00] R ≥ 50).

## 4 DISCUSSION

Over time, it has become accepted that venous pathology, and specifically chronic venous diseases such as varicose veins, develop as a result of increased pressure.^31^, ^32^ Current studies suggest that a set of factors act on this disease, causing changes in intrinsic structure and collagen content that play an important role in the aetiology of CVI.^31^, ^33^ Previous studies have shown interactions between different vein wall components, cells and especially collagen, demonstrating their indispensable role in the maintenance of venous tone and compression; moreover, ageing is known to produce changes in the vein wall structure.^34^ In this sense, our results indicate that valve incompetence increases ECM remodelling, manifested by alterations in COL‐I and COL‐III levels in the vein wall, and show that this process is significantly more remarkable in young patients. Many authors have published evidence of COL‐I and COL‐III coexpression and the alterations that occur in venous disease,^13^, ^31^, ^35^ but the present study is the first to report the relationships to valve incompetence. The data regarding altered collagen content in varicose veins are contradictory.^36^, ^37^ Xiao et al^38^ demonstrated an increase in collagen synthesis in cell cultures of smooth muscle varices compared with healthy controls. The multiple subtypes of collagen have a variety of functions, and veins are non‐linear viscoelastic structures. COL‐I tends to contribute the greatest mechanical stress to the rigid properties of veins at higher levels of distension, while COL‐III provides stress at lower levels of distension.^39^ The two collagen types have very similar biochemical compositions,^40^ but the presence of COL‐III could indicate different physiological processes. In our study, different collagen proteins (COL‐I and COL‐III) showed different gene expression profiles and histopathological expression patterns. Numerous studies on varicose veins have described the invasion of collagen fibres into both the tunica intima and tunica media, which lose their regular structure and adopt abnormal shapes.^41^ Histological studies have shown how the venous wall of patients with varicose veins suffer an alteration in the expression of COL‐I and COL‐III.^42^ Our data are in agreement with those of her authors but introduce the novelty of demonstrated changes in the ratio associated with valve incompetence. Along these lines, the histopathological studies of Gomez et al^43^ demonstrated how there was an increase in collagen fibres in patients with chronic venous disease. In addition, tissue demonstration studies have shown the importance of the expression of the FOXC2 gene in fibrosis suffered by the venous wall of patients with chronic venous disease.^44^

COL‐III has been shown to be necessary for distensible organs to reduce mechanical rigidity.^45^ Previous studies have shown that a decrease in the COL‐I/III ratio is indicative of the formation of heterotypic fibres, which have altered diameter and dimensions and thus affect organ functionality^46^, ^47^

This process could suggest possible distension of human veins under conditions of valve incompetence, depending on age. This finding supports the fact that young patients with venous reflux have a greater capacity for matrix remodelling. In this sense, these young patients with venous reflux show significantly higher MMP‐2 expression and significantly diminished TIMP‐2 expression. These results are consistent with those of numerous studies that have demonstrated MMP activity in venous disease.^15^, ^37^, ^48^ Previous researchers have stated that MMP‐2 is produced by both vascular and inflammatory cells.^18^, ^37^ The reported histological studies have shown how MMP activity is increased in the middle tunic of patients with chronic venous disease, which is in line with the trend of our results.^49^, ^50^ Serralheiro et al^49^ showed how the expression of the pro‐inflammatory components was increased in the tissue sample of these patients with chronic venous disease.

Metalloproteinases activity could be a compensatory mechanism in a pro‐inflammatory state with cellular damage caused by an alteration in venous flow. For the first time, our results show increased PARP and IL‐17 levels in the vein wall of patients with valve incompetence, which is remarkable in young patients. We can consider how the increases in these proteins lead to an increase in ECM fibres, which in the long term would compromise hydrostatic pressure. Henning et al^29^ reported PARP activity in vascular diseases as an immediate cellular response to DNA damage. Moreover, they proposed that vascular diseases increase cellular stress. Recent studies have shown that PARP is expressed in venous systems that suffer severe damage, such as calcification.^51^, ^52^ Csiszar et al^53^ noted the importance of PARP in endothelial dysfunction and ageing in the vascular system. In this sense, our previous studies demonstrated that patients with venous reflux have increased in molecules related to oxidative stress and lipid peroxidation, as well as increased activation of cellular pathways, such as the ERK pathway.^25^, ^26^ ERK has been shown to induce collagen fibre remodelling in pathological environments.^54^ Histological studies have revealed how ERK 1/2 protein expression was located with greater intensity in young patients with CVI and venous reflux.^54^ This fact is related to our results that show how young patients with venous reflux present the highest intensity of protein expression of cellular damage and inflammation, which correlates with the remodelling of collagen patterns from the histological point of view. In this sense, numerous histological studies have shown how the protein expression of transduction routes such as PI3K/Akt/mTOR is active in the three tunics of the venous wall of these patients with venous reflux, being significant in young patients with venous reflux.^26^ Furthermore, results of histological alterations have been reported in patients with CVI and molecules related to oxidative stress and lipid peroxidation.^27^ All these data make us think that CVI and venous reflux can be correlated with altered immunohistological patterns. From the histopathological point of view, the process could be considered as gradual; normal vein, failure, compensatory hypertrophy, atrophy and sclerosis. These histological changes can be seen in the studies reported by numerous authors in comparison with normal histological samples where they are not observed.^4^, ^25^, ^26^, ^27^, ^34^, ^42^, ^43^, ^44^, ^49^, ^50^

Thus, pro‐inflammatory environments have a prominent role in the cell damage process.^55^ Our results demonstrate that patients with valve incompetence have significantly increased IL‐17 levels, which is noteworthy in young patients. Buffa et al^56^ demonstrated that IL‐17 plays a prominent role in vascular disease and is a biomarker of damage and ageing. However, one of the limitations of our study is the need for protein studies using Western Blot technique with a study of larger sample size and age that allows a multivariate analysis to be carried out with comorbidities and specific pathophysiological changes, but we provide a detailed histological study of this protein expression.

## 5 CONCLUSIONS

Our findings indicate that patients with valve incompetence in the context of CVI undergo chronic expression of related protein with DNA damage, which has potential consequences not only at the local tissue level but also at the systemic level, possibly in association with ageing.

## CONFLICTS OF INTEREST

The authors confirm that there are no conflicts of interest.

## AUTHOR CONTRIBUTION

**Miguel A. Ortega:** Conceptualization (equal); Investigation (equal). **Oscar Fraile‐Martinez:** Investigation (equal). **Cielo García‐Montero:** Investigation (equal). **Leonel Pekarek:** Investigation (equal). **Miguel Alvarez‐Mon:** Investigation (equal). **Luis G. Guijarro:** Investigation (equal). **Maria del Carmen Boyano:** Investigation (equal). **Felipe Sainz:** Investigation (equal). **Melchor Álvarez‐Mon:** Conceptualization (equal); Funding acquisition (equal); Investigation (equal). **Julia Buján:** Investigation (equal). **Natalio García‐Honduvilla:** Investigation (equal). **Angel Asúnsolo:** Investigation (equal).

## Supporting information

Table S1

Table S2

## Data Availability

The data that support the finding of this study are available from the corresponding author upon reasonable request.
