# Supplementary figures and images for: Tissue remodelling and increased DNA damage in patients with incompetent valves in chronic venous insufficiency
Source: J Cell Mol Med. 2021 Jun 20;25(16):7878–89. doi: 10.1111/jcmm.16711 (PMC8358866; doi:10.1111/jcmm.16711)

## Slide 1
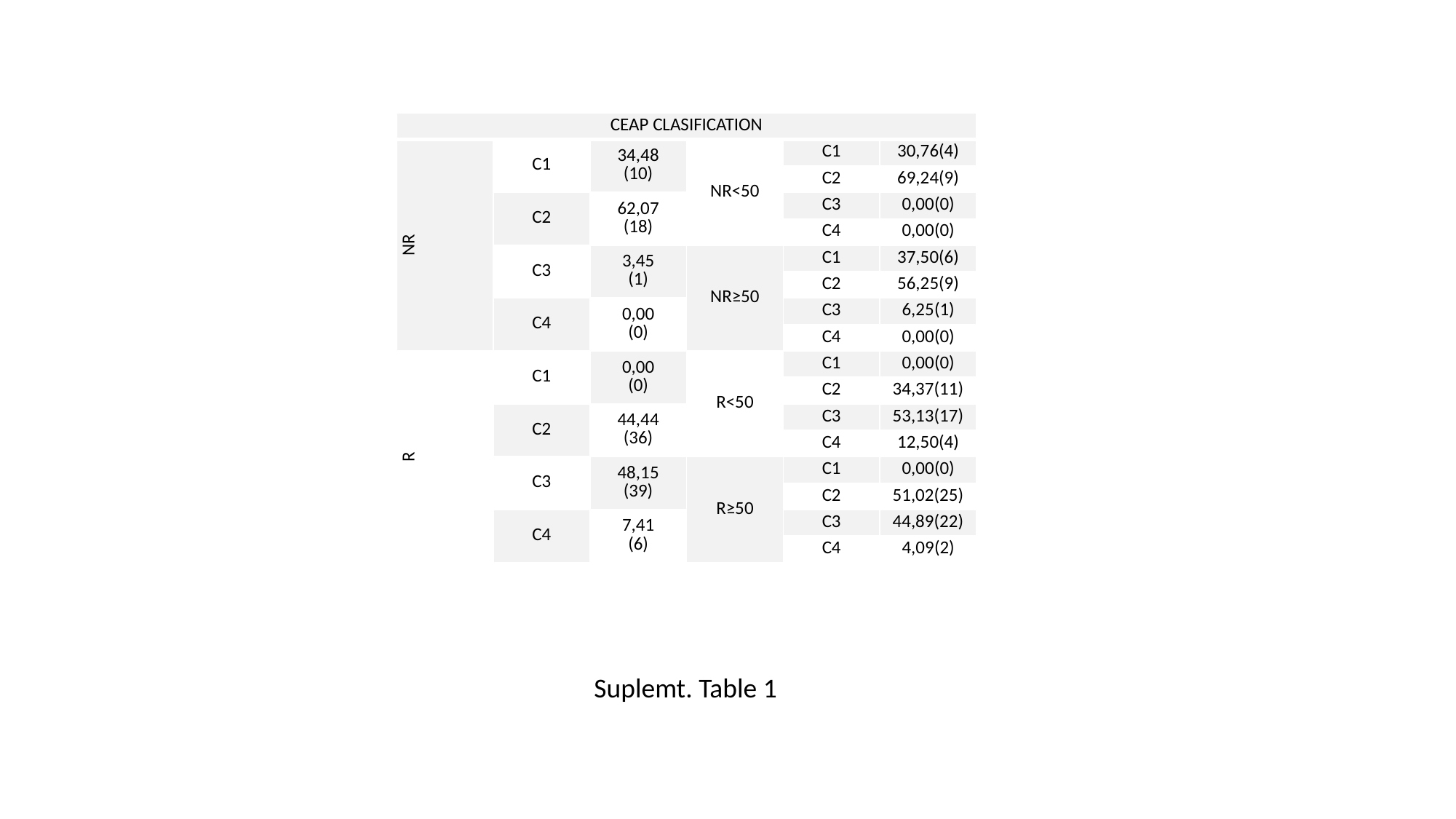

| CEAP CLASIFICATION | | | | | |
| --- | --- | --- | --- | --- | --- |
| NR | C1 | 34,48 (10) | NR<50 | C1 | 30,76(4) |
| | | | | C2 | 69,24(9) |
| | C2 | 62,07 (18) | | C3 | 0,00(0) |
| | | | | C4 | 0,00(0) |
| | C3 | 3,45 (1) | NR≥50 | C1 | 37,50(6) |
| | | | | C2 | 56,25(9) |
| | C4 | 0,00 (0) | | C3 | 6,25(1) |
| | | | | C4 | 0,00(0) |
| R | C1 | 0,00 (0) | R<50 | C1 | 0,00(0) |
| | | | | C2 | 34,37(11) |
| | C2 | 44,44 (36) | | C3 | 53,13(17) |
| | | | | C4 | 12,50(4) |
| | C3 | 48,15 (39) | R≥50 | C1 | 0,00(0) |
| | | | | C2 | 51,02(25) |
| | C4 | 7,41 (6) | | C3 | 44,89(22) |
| | | | | C4 | 4,09(2) |
Suplemt. Table 1

Supplement: Supplementary file 1 — Table S1 [file JCMM-25-7878-s001.pptx]
