# Supplementary material for: Tissue remodelling and increased DNA damage in patients with incompetent valves in chronic venous insufficiency
Source: J Cell Mol Med. 2021 Jun 20;25(16):7878–89. doi: 10.1111/jcmm.16711 (PMC8358866; doi:10.1111/jcmm.16711)
Supplement: Supplementary file 2 — Table S2 [file JCMM-25-7878-s002.pptx]

## Slide 1
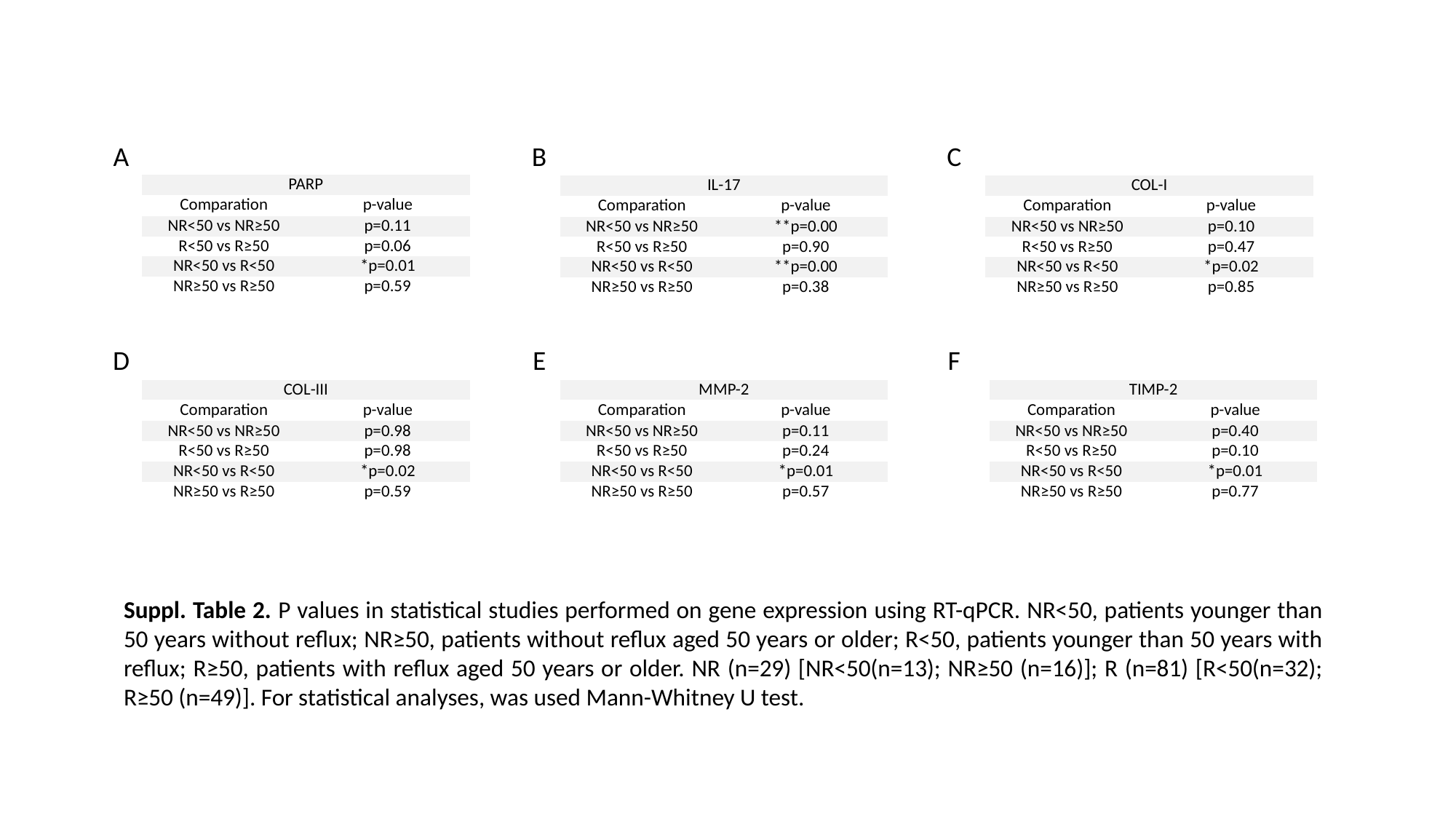

B
A
C
| PARP | |
| --- | --- |
| Comparation | p-value |
| NR<50 vs NR≥50 | p=0.11 |
| R<50 vs R≥50 | p=0.06 |
| NR<50 vs R<50 | \*p=0.01 |
| NR≥50 vs R≥50 | p=0.59 |
| IL-17 | |
| --- | --- |
| Comparation | p-value |
| NR<50 vs NR≥50 | \*\*p=0.00 |
| R<50 vs R≥50 | p=0.90 |
| NR<50 vs R<50 | \*\*p=0.00 |
| NR≥50 vs R≥50 | p=0.38 |
| COL-I | |
| --- | --- |
| Comparation | p-value |
| NR<50 vs NR≥50 | p=0.10 |
| R<50 vs R≥50 | p=0.47 |
| NR<50 vs R<50 | \*p=0.02 |
| NR≥50 vs R≥50 | p=0.85 |
E
D
F
| COL-III | |
| --- | --- |
| Comparation | p-value |
| NR<50 vs NR≥50 | p=0.98 |
| R<50 vs R≥50 | p=0.98 |
| NR<50 vs R<50 | \*p=0.02 |
| NR≥50 vs R≥50 | p=0.59 |
| MMP-2 | |
| --- | --- |
| Comparation | p-value |
| NR<50 vs NR≥50 | p=0.11 |
| R<50 vs R≥50 | p=0.24 |
| NR<50 vs R<50 | \*p=0.01 |
| NR≥50 vs R≥50 | p=0.57 |
| TIMP-2 | |
| --- | --- |
| Comparation | p-value |
| NR<50 vs NR≥50 | p=0.40 |
| R<50 vs R≥50 | p=0.10 |
| NR<50 vs R<50 | \*p=0.01 |
| NR≥50 vs R≥50 | p=0.77 |
Suppl. Table 2. P values in statistical studies performed on gene expression using RT-qPCR. NR<50, patients younger than 50 years without reflux; NR≥50, patients without reflux aged 50 years or older; R<50, patients younger than 50 years with reflux; R≥50, patients with reflux aged 50 years or older. NR (n=29) [NR<50(n=13); NR≥50 (n=16)]; R (n=81) [R<50(n=32); R≥50 (n=49)]. For statistical analyses, was used Mann-Whitney U test.
